# Mapping between HAQ-DI and EQ-5D-5L in a Chinese patient population

**DOI:** 10.1007/s11136-018-1925-1

**Published:** 2018-07-04

**Authors:** Thomas Patton, Hao Hu, Luan Luan, Keqin Yang, Shu-Chuen Li

**Affiliations:** 1 Centre for Health Economics, University of York, York, YO10 5DD UK; 2 School of Biomedical Sciences and Pharmacy, University of Newcastle, Callaghan, NSW Australia; 3 Department of Rheumatology, Taizhou No. 4 Renmin Hospital, Taizhou, Jiangsu China

**Keywords:** Mapping, Rheumatoid arthritis, Cost effectiveness, Health utilities

## Abstract

**Objectives:**

In order to address the current deficiency of health utility evidence relevant for economic evaluations involving treatments for rheumatoid arthritis (RA) in the Chinese setting, this study aims to develop a mapping algorithm linking the Health Assessment Questionnaire (HAQ) and EQ-5D-5L in a Chinese population of patients with RA.

**Methods:**

An estimation sample was obtained from a cross-sectional study that collected HAQ, the pain Visual Analogue Scale, and EQ-5D-5L in RA patients in two tertiary referral hospitals in China. Mapping algorithms were derived in this study using two alternative regression methods: the beta regression and a multivariate ordered probit regression. The internal validity of the mapping algorithms was assessed in each case by calculating predictive performance using a bootstrapping procedure.

**Results:**

Of the several algorithms developed using these data, predictive performance was shown to be better when VAS pain was included as a predictor and when the multivariate ordered probit regression method was used, rather than the beta regression method. The algorithms developed were shown to be comparable, in terms of predictive performance, to existing mapping studies despite the small sample size of the estimation data.

**Conclusion:**

It is hoped that the availability of these algorithms will facilitate the development of cost-effectiveness studies evaluating RA treatments in the Chinese health care setting.

**Electronic supplementary material:**

The online version of this article (10.1007/s11136-018-1925-1) contains supplementary material, which is available to authorized users.

## Introduction

Rheumatoid arthritis (RA) is a chronic, systemic inflammatory disorder and is primarily associated with progressive joint destruction and accompanied by pain, stiffness, and fatigue. If left untreated over the course of 10–20 years, RA may lead to significant disabilities and a severe reduction in the patient’s quality of life. The overall prevalence of RA is relatively constant across nations at 0.5–1% [1]. In China, RA is among the top 10 chronic diseases in terms of prevalence [2] and the second most common cause of disability [3]. Despite this heavy disease burden, the health care system in China does not currently cover biologic disease-modifying anti-rheumatic drugs (bDMARDs), which are an innovative class of therapeutic treatments for RA. This could potentially change however, as the reimbursement system in China looks set to move towards a value-based approach to health care coverage. In October 2010, the Ministry of Health (MoH) in China signed a memorandum of understanding with the National Institute for Health and Care Excellence (NICE) with a view to developing a new institution to promote quality and efficiency in health care. Furthermore, in an effort to facilitate future economic evaluations, Guidelines for Pharmacoeconomic Evaluations in China were published in 2011 [4]. These guidelines indicated a preference for the development of cost-effectiveness analyses (CEA) with quality-adjusted life years (QALY) using health utility data derived from preference-based, generic health-related quality of life (HRQL) instruments.

Although there have been two studies to date conducting CEA for RA treatments in China [5, 6], neither of these studies used health utility evidence that was relevant to the Chinese setting. It is not uncommon to find that clinical trials collect disease-specific (non-preference based) HRQL instruments, as opposed to generic preference-based instruments, capable of deriving health utility values directly. For other countries, such as the UK and Spain, studies are available which map between disease-specific instruments, e.g. the Health Assessment Questionnaire (HAQ) for RA [7], and generic preference-based measures of HRQoL, such as the EQ-5D [8], via statistical modelling [9–19]. Although this approach has its shortcomings, in the absence of directly relevant health utility data, it is often considered acceptable by the national reimbursement jurisdictions, for example NICE [20]. One of the existing CEA studies on RA treatments in China used a mapping algorithm but the values correspond to a tariff from another country [5]. Ideally, health utility values employed in CEA studies should reflect the preferences of the jurisdiction under investigation given that there are important differences between tariffs from different countries due to cultural differences [21], and this can have a substantial impact on the findings of a CEA study [22]. To the authors’ knowledge, there has yet to be any research conducted in a Chinese patient population to develop a mapping between HAQ and EQ-5D. In order to address the current deficiency of health utility evidence relevant for economic evaluation in the Chinese setting, the purpose of this study was to develop mapping algorithms linking the HAQ and EQ-5D-5L in a Chinese patient population. The authors anticipate that the availability of these algorithms will enable researchers to make use of the growing number of studies that have collected HAQ data in RA patients in China for the purposes of economic evaluation [23–26].

## Methods

### Data collection

A cross-sectional study was conducted in two tertiary referral hospitals, one in the Taizhou 5th Renmin Hospital in Jiangsu province and the other in Jianping County Hospital in Liaoning Province. The collection of data in separate regions of China was motivated by efforts to account for the significant heterogeneity, both in terms of economic development and living conditions, that exists across the Chinese population [27]. The study was approved by the Institutional Review Board and patients with RA were recruited, with informed consent, as a consecutive sample (i.e. data collection in all eligible patients until the desired sample size has been achieved) between May and December 2013. Patients were eligible if they satisfied the following inclusion criteria:

- they have been previously documented as experiencing RA;
- they were not affected by any other type of musculoskeletal problem;
- they were aged 18 years or older, and identified as being capable of completing self-reported HRQoL questionnaires in Chinese;
- they had no serious psychiatric disorder or cognitive dysfunction; and
- they were not a current substance abuser.

Eligible subjects were asked to complete HAQ and EQ-5D questionnaires by a trained interviewer, in addition to a series of questions pertaining to their socio-demographic characteristics. Symptom severity and functional limitations were measured in subjects by physicians using the ACR classification of Global Functional Status. The interview procedure—including the interviewer asking the questions—was identical in the two recruiting centres.

### Instruments

#### HAQ

The Health Assessment Questionnaire (HAQ), developed by Fries and colleagues in 1978 [28], has been widely administered and validated in patients for a range of rheumatic diseases. Moreover, it has become a de facto mandated outcome measure for clinical trials in rheumatoid arthritis as a component of the American College of Rheumatology criteria [29], which has subsequently been adopted for use in many RA studies in China [23–26, 30]. There were two components to the HAQ in this study: the HAQ Disability Index (HAQ-DI) and the HAQ pain visual analogue scale (VAS). The HAQ-DI consists of 20 items covering 8 domains assessing physical disabilities: dressing and grooming, arising, eating, walking, hygiene, reach, grip, and common daily activities. An additional 13 questions were included to assess the use of assistive devices in patients and a further 8 questions to assess whether or not patients received help from another person. Responses to each of the HAQ-DI questions are graded as follows: without any difficulty (0); with some difficulty (1); with much difficulty (2); and unable to do (3). The highest score for any component question in a category determines the category score. Two composite scores can be calculated, one with and one without the aids/devices element [27]. The HAQ pain VAS score is measured on a horizontal line where each end represents opposite ends of a continuum that is standardized to 15 centimeters in length. It is labelled with “no pain” and a score of 0 at one end, and “very severe pain” and a score of 100 at the other end. Patients are instructed to place a vertical mark on the line to indicate the severity of their pain. A score from 0 to 100 is obtained based on the location of the respondent’s mark.

#### EQ-5D-5L

EQ-5D-5L is a generic, preference-based HRQoL instrument with five dimensions including morbidity, self-care, usual activities, pain/discomfort, and anxiety/depression [31]. Each dimension has five response levels (no problems, slight problems, moderate problems, severe problems, and unable to/extreme problems) expressed by a 1-digit number, which results in a 5-digit number describing the respondent’s health state. Health utility index values were derived using a tariff taken from a recently developed study capturing health preferences in a sample of individuals from the general population in China [32]. The EQ-5D-5L was selected for use rather than the original EQ-5D instrument (EQ-5D-3L) on the grounds that the EQ-5D-5L was developed in an effort to improve on the original instrument’s perceived lack of sensitivity and also to reduce ceiling effects [31, 33].

### Statistical methods

A variety of statistical methods have been proposed in the published literature for the development of mapping algorithms [34]. Much of the debate in this area has revolved around the development of statistical methods capable of handling the unique distributional features of health utility values [35]. Ordinary least squares (OLS) regression methods are, at least in theory, inappropriate on the grounds that they do not guarantee predicted values will lie within a plausible range [36–38]. One proposed method for circumventing this problem is to employ regression estimators based on features of the beta distribution, which assumes that the dependent variable is restricted to a range of values between 0 and 1 [39, 40]. Another method known as the adjusted-limited dependent variable mixture model (ALDVMM) was developed to capture specific features of the UK version of the EQ-5D-3L tariff [38].

An alternative approach to mapping is to analyse the health state descriptions directly, rather than the health utility values, with modelling techniques including multinomial logit [41], ordered logistic [42], and ordered probit [43]. Unfortunately, these techniques fail to account for the correlations occurring between the different dimensions, which can give rise to misleading predictions. More recently, a study by Conigliani and colleagues showed that this issue is avoided by using a multivariate ordered probit method [44].

Mapping algorithms are derived in this study using two of the aforementioned regression methods: the beta regression method and a multivariate ordered probit regression. The ALDVMM approach is not employed in this study on the grounds that this method was developed for the purposes of analysing health utilities obtained using the UK version of the EQ-5D-3L tariff. Although there are additional analytical methods beyond those discussed in this paper, the methods selected have been identified in ongoing research as holding the most potential for the purposes of mapping [45]. All analyses were undertaken in R, in which the beta regression method was implemented the ‘betareg’ package [46] and the multivariate approach was implemented using the ‘mvord’ package [47]. The index values of the EQ-5D-5L, which ranged between − 0.391 and 1, had to be rescaled given that the beta regression handles values lying between 0 and 1. The following equation was used to rescale values:

$$rescaled\;value=(original\;value+0.391)/1.391.$$

Moreover, values lying at the either end of the distribution needed to be adjusted given that the beta regression cannot accommodate values of 0 or 1. As such, 1e-12 was added to values equal to 0 and subtracted from values equal to 1.

### Choice of predictors

There are multiple ways in which the HAQ could be incorporated into a mapping algorithm. The simplest approach would be to regress the overall HAQ score onto the EQ-5D. Alternatively, one could either use the HAQ item responses (i.e. questionnaire responses) or the subdomain scores (e.g. dressing, arising). Although the latter methods offer greater detail in the description of HRQoL effects, they may not be convenient for practical purposes. For instance, suppose that one wanted to predict health utility values using aggregated HAQ scores from a published study; mapping algorithms derived using item responses or subdomain scores would be incompatible with this evidence. Furthermore, the authors also felt that the overall HAQ score would be a more appropriate predictor given the small sample size of the estimation dataset (i.e. fewer parameters requiring estimation). Supplementary analyses are conducted with VAS pain score as an additional predictor following recommendations from Madan and colleagues [48].

### Validation of mapping algorithms

The internal validity of the mapping algorithms is explored using bootstrapping procedures. First, a bootstrap sample is drawn from the original estimation dataset, with replacement and a sample size equal to that of the original dataset. Mapping algorithms are then derived from the bootstrap sample, for each of the proposed modelling approaches. The estimated algorithms are then used to predict health utility values in the original estimation dataset. Finally, the resulting predictions are compared to the observed health utility values using the root mean squared error (RMSE) statistic term. This process is repeated until 500 bootstrap iterations have been run.

When making predictions using the MV Probit approach, probabilities are required for each of the relevant health state descriptions. In theory, this would require predictions for each of the 3125 feasible states (5^5) that can be constructed using the health state description of the EQ-5D-5L given that the MV Probit model accounts for correlations between each of the five dimensions. Thankfully, this is not necessary given that full health is the only state where between-dimension correlations have an impact on the index values derived using the Chinese EQ-5D-5L tariff. Consequently, the remaining probabilities reflect responses to specific dimensions, independent of the other dimensions. Once the probabilities have been predicted, EQ-5D values can then be scored. The authors decided to use the ‘expected utility’ method proposed in a study by Le and Doctor given that this ensures an exact calculation via an algebraic equation [49]. The following equation was used to conduct these calculations:

$$\begin{aligned}EQ5D\;prediction=&\,\left( {Pro{b_{Full\;Health}}} \right) \times 1+(1 - Pro{b_{Full\;Health}}) \times (1 - dis) \\ dis =&\,\{ (Pro{b_{AD,2}} \times 0.258)+(Pro{b_{MB,2}} \times 0.345)+(Pro{b_{PD,2}} \times 0.302) \\ &+(Pro{b_{SC,2}} \times 0.253)+(Pro{b_{UA,2}} \times 0.233)\} \times 0.191 \\ & +\{ (Pro{b_{AD,3}} \times 0.258)+(Pro{b_{MB,3}} \times 0.345)+(Pro{b_{PD,3}} \times 0.302) \\ &+(Pro{b_{SC,3}} \times 0.253)+(Pro{b_{UA,3}} \times 0.233)\} \times 0.458 \\ &+\{ (Pro{b_{AD,4}} \times 0.258)+(Pro{b_{MB,4}} \times 0.345)+(Pro{b_{PD,4}} \times 0.302) \\ &+(Pro{b_{SC,4}} \times 0.253)+(Pro{b_{UA,4}} \times 0.233)\} \times 0.832 \\ &+\{ (Pro{b_{AD,5}} \times 0.258)+(Pro{b_{MB,5}} \times 0.345)+(Pro{b_{PD,5}} \times 0.302) \\ &+(Pro{b_{SC,4}} \times 0.253)+(Pro{b_{UA,4}} \times 0.233)\}, \end{aligned}$$

where ${Prob}_{Full Health}$ represents the probability of a full health response and ${Prob}_{i,j}$ represents the probability of a response of *j* on dimension *i*.

## Results

### Descriptive statistics and missing data

Table 1 provides descriptive statistics for the estimation sample used to derive mapping algorithms. The EQ-5D-5L responses in the complete case sample, without VAS pain scores (*n* = 130), covered 43 of the 3,125 health states. The same finding was observed in the complete case sample with VAS pain scores (*n* = 125). The proportion of patients reporting no health problems on any of the dimensions of the EQ-5D-5L was below 2% in the sample without VAS pain scores (2/130) and less than 1% in the sample with VAS pain scores (1/125).

**Table 1:** Descriptive statistics for the estimation sample

	Original data (*n* = 133)		Complete sample, without VAS pain (*n* = 130)	Complete sample, with VAS pain (n = 125)
	Mean (SD)	Missing data (%)	Mean (SD)	Mean (SD)
EQ-5D	0.66 (0.38)	1/133 (> 1%)	0.67 (0.38)	0.67 (0.37)
HAQ	1.22 (0.86)	2/133 (> 2%)	1.20 (0.86)	1.23 (0.84)
VAS pain	50.3 (24.6)	5/133 (> 4%)	–	50.0 (24.8)

Overall, the degree of missing data in the predictor variables was small, thus reducing the concerns one might have about obtaining less precise parameter estimates in a complete case analysis (i.e. due to reduced statistical power). In order to examine the validity of assuming that the data are missing completely at random (MCAR), logistic regressions were performed to explore any associations between the probability of a given predictor being missing and the values observed in the other predictors. No statistically significant associations were observed. This approach follows recommendations relating to the methods for best practice in the development of prediction models [50]. Additional tests found no statistically significant associations between the probability of a given predictor being missing and the values observed in other auxiliary variables (health care setting and region). In view of these findings, the authors decided that the risks posed by the missing data in the estimation sample were minimal and that a complete case analysis would be reasonable.

### Model estimates

Parameter estimates, alongside associated standard errors, for each of the mapping algorithms can be found in the supplementary materials. R scripts can also be found in the supplementary materials to implement the algorithms in mapping applications. This includes Cholesky decomposition matrices corresponding to each of the models. Models 1a and 2a refer to algorithms developed with HAQ score as the only covariate, while models 1b and 2b refer to the algorithms developed with both HAQ score and pain covariates. Note that predictions derived using the beta regression models need to be converted back onto the original scale between − 0.391 and 1 using the following equation:

$$original~scale=\left( {predictions~ \times ~1.391} \right) - 0.391.$$

### Validation of mapping algorithms

Table 2 contains the results from the bootstrapping procedures used to test the internal validity of the mapping algorithms. The MV Probit approach exhibited a lower RMSE in the majority of the bootstrap samples when compared to the beta regression approach. We can, therefore, conclude that the MV Probit modelling approach has a stronger predictive performance for the estimation sample under investigation when compared to the beta regression approach. The results also demonstrate that predictive performance is improved when the VAS pain covariate is included. Table 2 also shows that predictive performance is better in HAQ score less than 1 compared to those above. This finding is consistent with the broader mapping literature [35], i.e. that predictive performance is worse in poorer health states.

**Table 2:** Internal validation of mapping algorithms

	All patients	Patients with HAQ less than 1	Patients with HAQ between 1 and 2	Patients with HAQ greater than 2
Models with HAQ covariate only				
RMSE (SD)				
MV probit	0.196 (0.013)	0.065 (0.003)	0.300 (0.019)	0.293 (0.039)
Beta	0.246 (0.021)	0.094 (0.018)	0.376 (0.037)	0.343 (0.011)
Samples where RMSE was lower in the MV probit (%)	100	99.6	99.8	91.8
Models with HAQ and VAS pain covariates				
RMSE (SD)				
MV probit	0.177 (0.022)	0.088 (0.022)	0.226 (0.027)	0.277 (0.031)
Beta	0.196 (0.016)	0.099 (0.014)	0.261 (0.028)	0.281 (0.014)
Samples where RMSE was lower in the MV probit (%)	94.2	90.8	95.2	63.8

### Applying the preferred mapping algorithm using R code

The results of the bootstrapping exercise showed that the optimal mapping algorithm is model 1b, i.e. MV Probit method with both HAQ score and pain covariates, due to it having the best predictive performance of the four alternatives. There are several stages involved in the prediction of EQ-5D values using this algorithm and these have been set out in an R script, along with a corresponding example, in the supplementary materials (see ‘Supplementary-Materials-2.docx’). This code has been annotated to demonstrate how the algorithm can be implemented in the R software package and how it can be adapted for the purposes of mapping applications involving data containing HAQ and VAS pain measures.

In the first stage of the code, the user is asked to specify whether the predicted values will be used to obtain deterministic or probabilistic cost-effectiveness results. In the case of the former, point estimates associated with each of the mapping parameters (i.e. regression coefficients, threshold values, and the error structure) will be used in the subsequent stages. Alternatively, if inputs for a probabilistic cost-effectiveness analysis are required, predictions will be made using parameter estimates that are randomly selected from a distribution that reflects the sample uncertainty surrounding the point estimates of each of the mapping parameters. In addition, the user is asked to load HAQ and VAS pain data to predict EQ-5D values (example data have been provided for the purposes of demonstration).

Stages 2 and 3 of the R script load the parameter estimates associated with model 1b, along with the corresponding variance–covariance matrix, which is used to construct sample distributions for each of the parameters. In stage 4, probabilities associated with different responses to items of the EQ-5D-5L are predicted. The code in stage 4 is based on the get.prob() function from the *mvord* package and has been adapted to accommodate external data. The final stage of the R script calculates the expected EQ-5D values by combining the probabilities derived in the previous step with the weights from the tariff estimated by Luo, Liu, and colleagues [32].

## Discussion

This study is the first to develop mapping algorithms linking HAQ scores to a preference-based measure of HRQoL in a Chinese patient population. Until now, the absence of such evidence has been a major obstacle for researchers seeking to find relevant evidence to populate CEAs comparing RA treatments in China. The availability of the algorithms in this study is timely given that there has been an increasing number of CEAs conducted in the Chinese health care setting [51]. Moreover, recent actions by the Ministry of Health in China point towards an increased use of HTA for policy-making purposes [52, 53]. It is hoped that the availability of R scripts for the implementation of the algorithms will facilitate their usage in applied CEA studies.

The mapping algorithms were derived using a cross-sectional dataset conducted in two hospitals in China that collected EQ-5D-5L, HAQ scores, and VAS pain scores in patients with RA. Two established statistical methodologies—the beta regression and the MV ordered probit regression—were employed to develop mapping algorithms. These fundamentally different approaches to mapping were selected in light of ongoing research identifying them as holding the most potential for overcoming the well-established shortcomings associated with standard OLS regression methods for the purposes of mapping.

The predictive performance of the mapping algorithms developed in this study was tested by estimating the RMSE for each model specification using a bootstrapping procedure. Using this approach, the MV ordered probit model exhibited lower prediction errors when compared to the beta regression model. Prediction errors were also lower in those models including a VAS pain covariate. Overall, the predictive performance of the MV ordered probit models was consistent with the range of RMSEs (0.1644–0.207) observed in mapping studies for RA in the published literature [11, 16, 18, 19, 38, 43]. To the authors’ knowledge, this study is the first to compare the predictive performance of the beta and MV ordered probit methods for the purposes of mapping in cost-effectiveness applications. In this study, the computational burden incurred when predicting EQ-5D values using the MV ordered probit method was not as extensive as it could potentially have been. This was because, aside from the full health state, between-dimension correlations did not have an impact on the resulting values obtained when applying the Chinese EQ-5D-5L tariff. It is important to acknowledge that a different approach may have been needed for a tariff with a different model specification.

It is important to recognize that there are several limitations associated with the evidence used in this study. The sample size of the dataset used to derive the mapping algorithms was small, with only 130 patients (125 in models including the VAS pain as a predictor), in comparison to similar studies in the published literature [9–19]. A larger sample would be preferable given that an increase in statistical power leads to improved precision in the estimation of parameters. Ultimately, this is important in the context of a decision model as it can potentially result in reduced parameter uncertainty in the cost-effectiveness estimates. Although the bootstrapping exercise in this study provides an assessment of the internal validity of the mapping procedures considered in this study, it does not tell us anything about the generalizability (or external validity) of the algorithms. Ideally, researchers should investigate the generalizability of prediction models using an independent dataset [50]. Unfortunately, the lack of external data meant that the authors were unable to do this.

Another potential limitation in this study is the exclusion of patients with other muscoskeletal problems, which may confound the relationship between HAQ scores and EQ-5D values. The degree to which this may ultimately affect cost-effectiveness results for a given research question will depend on the prevalence of other muscoskeletal comorbidities in the patient population under investigation. Researchers should yield caution when applying the mapping algorithms from this paper if the prevalence of such comorbidities is high.

There are various ways in which the HAQ data could have been specified in terms of predictors included in the mapping algorithms. Some previous studies have captured HAQ data in the form of categorical responses to the questionnaire items using dummy variables [11, 13, 16]. Backwards or forwards stepwise selection procedures are typically used to identify significant items rather than using all 42 individual items. Another approach has been to specific predictors reflecting the 8 domains within the HAQ measure [11], i.e. dressing/grooming, rising, eating, walking, hygiene, reach, grip, activities. One advantage of specifying HAQ data in terms of item-level responses or domain-level scores, rather than overall index scores, is that they have the potential to account for a higher proportion of the variance in the dependent variable. However, mapping algorithms containing item-level responses as predictors can only be used to predict EQ-5D values in conjunction with HAQ data at the individual patient level; consequently, this rules out the possibility of using evidence from the published literature. Moreover, the specification of HAQ data in terms of item-level responses or domain-level scores implies a larger number of predictors and, consequently, a reduction in the statistical power for a given sample size. The overall HAQ score was considered to be the most appropriate predictor for the purposes of this study given the small sample size of the estimation dataset (i.e. fewer parameters requiring estimation).

## Conclusions

In recent years, the availability of mapping algorithms linking HAQ to generic measures of HRQL has facilitated the development of cost-effectiveness studies evaluating treatments for RA [20]. This study is the first, to the authors’ knowledge, to have developed a mapping algorithm between HAQ and EQ-5D in a Chinese patient population. The estimation sample was obtained form a cross-sectional study that collected data in RA patients in two tertiary referral hospitals in China. Of the several algorithms developed using these data, predictive performance was shown to be better when VAS pain was included as a predictor and when the multivariate ordered probit regression method was used, rather than the beta regression method. The algorithms developed were shown to be comparable, in terms of predictive performance, to existing mapping studies despite the small sample size of the estimation data. It is hoped that the availability of these algorithms will facilitate the development of cost-effectiveness studies evaluating RA treatments in the Chinese health care setting.

## Electronic supplementary material

Below is the link to the electronic supplementary material.

Supplementary material 1 (DOCX 121 KB)

Supplementary material 2 (DOCX 129 KB)
