# Supplementary material for: Mapping between HAQ-DI and EQ-5D-5L in a Chinese patient population
Source: Qual Life Res. 2018 Jul 4;27(11):2815–22. doi: 10.1007/s11136-018-1925-1 (PMC6208580; doi:10.1007/s11136-018-1925-1)
Supplement: Supplementary file 1 — Supplementary material 1 (DOCX 121 KB) [file 11136_2018_1925_MOESM1_ESM.docx]

Supplementary Table 1: Multivariate Ordered Probit Results (Coefficients and Threshold Values)

|  | Estimate (SE) | |
| --- | --- | --- |
|  | Model 1a | Model 1b |
| Mobility ~ HAQ score | 1.139 (0.172) | 0.972 (0.188) |
| Mobility ~ Painscale |  | 0.015 (0.006) |
| Self Care ~ HAQ score | 1.289 (0.195) | 1.088 (0.223) |
| Self Care ~ Painscale |  | 0.022 (0.007) |
| Usual Activities ~ HAQ score | 1.123 (0.167) | 0.961 (0.186) |
| Usual Activities ~ Painscale |  | 0.014 (0.006) |
| Pain ~ HAQ score | 0.860 (0.154) | 0.612 (0.190) |
| Pain ~ Painscale |  | 0.023 (0.007) |
| Anxiety ~ HAQ score | 0.923 (0.147) | 0.731 (0.164) |
| Anxiety ~ Painscale |  | 0.018 (0.006) |
| Mobility \| Threshold 1 | 0.632 (0.220) | 1.115 (0.326) |
| Mobility \| Threshold 2 | 2.179 (0.262) | 2.759 (0.394) |
| Mobility \| Threshold 3 | 2.812 (0.340) | 3.462 (0.463) |
| Mobility \| Threshold 4 | 3.743 (0.511) | 4.500 (0.642) |
| Self Care \| Threshold 1 | 2.049 (0.292) | 2.895 (0.417) |
| Self Care \| Threshold 2 | 2.812 (0.382) | 3.838 (0.569) |
| Self Care \| Threshold 3 | 3.286 (0.390) | 4.395 (0.596) |
| Self Care \| Threshold 4 | 4.092 (0.588) | 5.321 (0.759) |
| Usual Activities \| Threshold 1 | 1.309 (0.227) | 1.782 (0.312) |
| Usual Activities \| Threshold 2 | 2.148 (0.265) | 2.676 (0.378) |
| Usual Activities \| Threshold 3 | 2.936 (0.358) | 3.529 (0.467) |
| Usual Activities \| Threshold 4 | 3.487 (0.368) | 4.134 (0.449) |
| Pain \| Threshold 1 | -1.283 (0.313) | -0.639 (0.460) |
| Pain \| Threshold 2 | 1.513 (0.244) | 2.396 (0.378) |
| Pain \| Threshold 3 | 2.200 (0.293) | 3.231 (0.426) |
| Pain \| Threshold 4 | 3.153 (0.400) | 4.341 (0.465) |
| Anxiety \| Threshold 1 | 0.990 (0.202) | 1.597 (0.282) |
| Anxiety \| Threshold 2 | 1.900 (0.249) | 2.607 (0.374) |
| Anxiety \| Threshold 3 | 2.850 (0.357) | 3.701 (0.442) |
| Anxiety \| Threshold 4 | 3.376 (0.385) | 4.290 (0.429) |

Supplementary Table 2: Multivariate Error Structure for Model 1a

|  | Anxiety | Mobility | Pain | Self-care | Usual Activities |
| --- | --- | --- | --- | --- | --- |
| Anxiety | 1.000 | 0.752 | 0.829 | 0.833 | 0.822 |
| Mobility | 0.752 | 1.000 | 0.688 | 0.893 | 0.907 |
| Pain | 0.829 | 0.688 | 1.000 | 0.729 | 0.729 |
| Self-care | 0.833 | 0.893 | 0.729 | 1.000 | 0.916 |
| Usual Activities | 0.822 | 0.907 | 0.729 | 0.916 | 1.000 |

Supplementary Table 3: Multivariate Error Structure for Model 1b

|  | Anxiety | Mobility | Pain | Self-care | Usual Activities |
| --- | --- | --- | --- | --- | --- |
| Anxiety | 1.000 | 0.644 | 0.791 | 0.770 | 0.828 |
| Mobility | 0.644 | 1.000 | 0.663 | 0.860 | 0.905 |
| Pain | 0.791 | 0.663 | 1.000 | 0.644 | 0.595 |
| Self-care | 0.770 | 0.860 | 0.644 | 1.000 | 0.894 |
| Usual Activities | 0.828 | 0.905 | 0.595 | 0.894 | 1.000 |

Supplementary Table 4: Beta Regression Results

|  | Estimate (SE) | |
| --- | --- | --- |
|  | Model 2a | Model 2b |
| Intercept | 2.597 (0.196) | 3.732 (0.248) |
| HAQ | -1.459 (0.125) | -1.164 (0.122) |
| Painscale |  | -0.025 (0.004) |
