# Supplementary material for: Mapping between HAQ-DI and EQ-5D-5L in a Chinese patient population
Source: Qual Life Res. 2018 Jul 4;27(11):2815–22. doi: 10.1007/s11136-018-1925-1 (PMC6208580; doi:10.1007/s11136-018-1925-1)
Supplement: Supplementary file 2 — Supplementary material 2 (DOCX 129 KB) [file 11136_2018_1925_MOESM2_ESM.docx]

# The term below clears all objects in the work environment

# rm(list=ls(all=T))

################################################################################

### Stage 1 (Model 1b): User Specifications

################################################################################

# The code below specifies whether predictions will be deterministic or

# probabilistic

probabilistic <- 1 # probabilistic = 1 ; deterministic = 0

# Need to create an object containing HAQ and VAS pain data and label this as

# 'predictors'. An example has been provided below containing made-up data

# points

predictors <- matrix(data=c(c(0.375, 1.625, 2.125, 1.625, 3.000),

c(60, 55, 40, 30, 65)),

nrow=5,ncol=2,dimnames = list(NULL,c("haq1","painscale")))

################################################################################

### Stage 2 (Model 1b): enter parameter point estimates

################################################################################

# sigma is the error structure for model 1b

sigma <- matrix(

data = c(1.0000000,0.6437712,0.7912376,0.7703542,0.8283033,0.6437712,

1.0000000,0.6627683,0.8595744,0.905238,0.7912376,0.6627683,

1.0000000,0.6439538,0.5946516,0.7703542,0.8595744,0.6439538,

1.0000000,0.8944501,0.8283033,0.905238,0.5946516,0.8944501,

1.0000000),

nrow = 5, ncol = 5, byrow = TRUE,

dimnames = list(c("ad","mo","pd","sc","ua"),

c("ad","mo","pd","sc","ua")))

# coefficients is the regression coefficients for model 1b

coefficients <- c(0.73055820, 0.97200502, 0.61191017, 1.08819221, 0.96146978,

0.01786853, 0.01498625, 0.02299354, 0.02186020, 0.01381784)

# theta is threshold values for model 1b

theta <- list(c(1.597304, 2.606510, 3.701212, 4.289984),

c(1.115285, 2.759396, 3.462357, 4.500355),

c(-0.6394355, 2.3960214, 3.2310568, 4.3407331),

c(2.895494, 3.837782, 4.395331, 5.321474),

c(1.781658, 2.675991, 3.528780, 4.133645))

################################################################################

### Step 3 (Model 1b): derive probabilistic estimates (only if probabilistic = 1)

################################################################################

# first need to load the variance covariance matrix for model 1b can be loaded

var_cov_1b <- matrix(data=

c(0.0792747685767784, 0.0894528478058515, 0.0948338110967241, 0.0830798208656088, 0.0470704715452998, 0.0517316269681289, 0.0553644656095927, 0.0549989491138828, 0.0575914564285976, 0.0591692689734905, 0.0618768677474458, 0.0608445552931662, 0.0540288483709562, 0.0619828922843429, 0.0631140319474451, 0.058772779019521, 0.0555658336989993, 0.0614511294772112, 0.0641548488776596, 0.0612881879700699, 0.0112006963283243, 0.00961279618616506, 0.00782461531119608, 0.00921422224757176, 0.00837842914724227, 0.00112663340003969, 0.00064075218581083, 0.000838141109400355, 0.000742435524876819, 0.000793761360187203, -0.00133930573499503, -0.000497848909223294, 0.000789816506243978, -0.00118563096723419, -0.00207735259778369, -0.00111751680135664, 0.000149852559388663, -0.00149219667391316, -0.00113488560297221, -0.000200278213983808,

0.089452847805848, 0.140176738349028, 0.132007819318778, 0.113027274036985, 0.0507775953071723, 0.0608954680716884, 0.0636803529141394, 0.0670201766321981, 0.0610128882385825, 0.0740854006721573, 0.0820632009595525, 0.078208587237133, 0.0632589711166462, 0.0800186692796034, 0.0813112087210481, 0.0753451254245529, 0.0622125231673156, 0.0761296075375685, 0.078826747535324, 0.0767731449022404, 0.0114242086825814, 0.0137893469782209, 0.00934883549715374, 0.0105552827567414, 0.0134736696483166, 0.0015166846628883, 0.000668686824332116, 0.0010009451782965, 0.000894722909358136, 0.00081588598460722, -0.00298481506342635, -0.00379229437967456, -0.00015389296207339, -0.00436134943948971, -0.00481974992215366, -0.000375511394176023, 0.0013121133714035, -0.00296326635195809, -0.00292991341196707, -4.43077964761828e-05,

0.0948338110967251, 0.132007819318774, 0.19559123073178, 0.159839950286286, 0.0515247843253703, 0.0642545960477331, 0.083294397185478, 0.100636159824471, 0.0619310326109187, 0.072265924567299, 0.0876681437449866, 0.104002214972869, 0.074103933866152, 0.0967259878871254, 0.105766875721275, 0.120064944210454, 0.0626301178004852, 0.0786805763223995, 0.101112507536064, 0.101362605963154, 0.0259908881385261, 0.0222938607875473, 0.0132034058657462, 0.0180595074953342, 0.019869626113996, 0.00143381924453272, 0.000583887643969917, 0.00100009201059687, 0.00100186238932449, 0.000811141869221623, -0.00771449830405061, -0.00541322334905796, -0.00823426369711037, -0.00539218716136588, -0.00381782689489426, -0.00434435112911258, -0.00173225984370867, -0.00235439262059474, -0.00255151662082289, -0.00334040119800356,

0.0830798208656151, 0.113027274036992, 0.159839950286278, 0.183811248267157, 0.0492335910790164, 0.0621267557325343, 0.0835199124513889, 0.105498532708033, 0.0648619662780002, 0.0742902583765081, 0.0873193439228514, 0.128066936456603, 0.0654923164116948, 0.0862799317418908, 0.0958389800419094, 0.107689293405164, 0.0607989650126607, 0.077245457621917, 0.100172386692149, 0.110455385528053, 0.0267111553302112, 0.0233556272960893, 0.0154930725558283, 0.01748493619253, 0.0209492699125735, 0.0011872710301634, 0.000557789514086167, 0.000997419267902923, 0.000857741991673173, 0.000801909567185781, -0.00748741357277952, -0.00549826852062595, -0.00727935962739034, -0.00561515174896808, -0.00505491061038954, -0.00632141927926306, -0.00239524619341638, -0.00317135815187834, -0.00254708842935947, -0.00406856559643957,

0.047070471545307, 0.0507775953071983, 0.0515247843253863, 0.0492335910789974, 0.106167242916246, 0.109153666927831, 0.121993708288585, 0.149716142911633, 0.0487145888811413, 0.0500518417876722, 0.0557886563932468, 0.0448550876486537, 0.0686127239956467, 0.0684145376240247, 0.0761415619958625, 0.0852354584732169, 0.0712318797122806, 0.0762040877550142, 0.0786040582730615, 0.0827520444382598, 0.00763426281829046, 0.0231314472853515, 0.0060742314972579, 0.0130228439039867, 0.014256274790834, 0.000682446551014167, 0.00133827668224226, 0.000715172727819506, 0.000893115634793116, 0.00096518831449332, -0.00208065478519284, -0.000673363649259058, -0.00657020355986599, -0.000352332962435028, -0.000394509568454031, -0.00230687185109435, -0.00225379706937031, -0.000639983059222061, 0.00100817289209153, -0.00222457604061802,

0.0517316269681407, 0.060895468071711, 0.0642545960477502, 0.062126755732534, 0.10915366692783, 0.155407541567814, 0.165408403981515, 0.199625042581485, 0.0501515642060084, 0.063111544858232, 0.0697405296684727, 0.0549410190377002, 0.0932041929547228, 0.102102008516138, 0.113766168130714, 0.130202024444095, 0.0824671224596437, 0.1013675047325, 0.106966338018313, 0.113028470156399, 0.0108154213009765, 0.0299971924720941, 0.00553500598291485, 0.0213776012317404, 0.0203063142886058, 0.000703612983918376, 0.00164545291942402, 0.000842953633599491, 0.00109634007814396, 0.00111541811646093, -0.00615899283340982, -0.000918898327171107, -0.0107220997568324, -0.0019329966621173, -0.00599550169747934, -0.00485499030221154, -0.00505054844487281, -0.00140568607572529, 0.00130107434513138, -0.00432850981824554,

0.0553644656096094, 0.0636803529141493, 0.0832943971854948, 0.0835199124514202, 0.12199370828859, 0.165408403981518, 0.214433436375093, 0.253002956604781, 0.0488166055217562, 0.0673348500417778, 0.0816017043731479, 0.0647805241463824, 0.0979268907730291, 0.112180954038351, 0.134175921025086, 0.15528921404286, 0.087670107679943, 0.110732590749, 0.131039484851338, 0.139391078896391, 0.0167052729480304, 0.043849076697351, 0.00672250388218019, 0.0285859844153035, 0.025150212684459, 0.000731730760982666, 0.0017094259763254, 0.00089748889515557, 0.00106803539330156, 0.00118251237624367, -0.0104195078240248, -0.00146003878922382, -0.0127638582988782, -0.00354902449956869, -0.00776269257226532, -0.00945089478487617, -0.00751525518607426, -0.00252224039965052, 0.000550359773863934, -0.00608511348814487,

0.054998949113904, 0.0670201766322013, 0.100636159824491, 0.105498532708092, 0.149716142911642, 0.199625042581491, 0.253002956604789, 0.412719537713957, 0.043827012590619, 0.0678499994504155, 0.0806824836806517, 0.0685351372233949, 0.113520959160075, 0.131729771972842, 0.164771190917266, 0.234101479853799, 0.0885724473629671, 0.11150963895215, 0.135616283976314, 0.170490968111431, 0.0242979064656298, 0.0679713970039142, 0.0113794484971488, 0.0379632059569411, 0.0326933593555034, 0.000605746874660208, 0.00187300878697375, 0.000832115075863381, 0.00120822934274999, 0.00113432287578053, -0.0148285766963702, -0.000879486994537729, -0.0231374327825341, -0.00531231659307465, -0.00998871836530623, -0.0124469157575294, -0.00993965956814519, -0.00365997145496519, 0.00135998034527086, -0.0115706757465216,

0.0575914564285939, 0.0610128882385774, 0.0619310326109103, 0.0648619662779979, 0.0487145888811381, 0.0501515642059991, 0.0488166055217373, 0.0438270125905957, 0.211706228323379, 0.129335779961175, 0.140143018506734, 0.141303324531502, 0.045277476721958, 0.0470405974515798, 0.0435535345797544, 0.0270873529989133, 0.0618401472590815, 0.0651968913342623, 0.0660902265535381, 0.0663800061981824, 0.00813233281744072, 0.00617348485206911, 0.0169717479897898, 0.0013070575559808, 0.00967825339586873, 0.000961224342272461, 0.000754678784243154, 0.0020692542481607, 0.000910279726520193, 0.001008681661875, 0.00130251077532984, -0.000541963706084025, -0.00530097743907929, -0.000128799873373248, 0.00207249105543964, 0.00101623827640015, 0.00181663003374076, 0.0063804518723708, 0.00615250158217924, 0.00174512170572596,

0.0591692689734861, 0.0740854006721523, 0.0722659245672893, 0.0742902583765017, 0.0500518417876654, 0.063111544858221, 0.0673348500417606, 0.0678499994503937, 0.129335779961174, 0.142691806658674, 0.14363855471834, 0.134285751709489, 0.0502154371589412, 0.0499121116119846, 0.0499550469062202, 0.0324719667340443, 0.0603817713220249, 0.0697953748435138, 0.0721582859464832, 0.0741303629456477, 0.00953578499131376, 0.0112337464420071, 0.0153174042247213, 0.00363334587089031, 0.0101969201783186, 0.000944208399448661, 0.000783984843067933, 0.00186932725795986, 0.000828205555254809, 0.0009396829268806, -0.00282121235614747, -0.00554113859624216, -0.00424292917768981, -0.00297879005007003, -0.00774324488350183, -0.000167531084233807, 0.000663795942712869, -0.00456032716790163, -0.00414611327679682, -0.000416742948960839,

0.0618768677474442, 0.0820632009595355, 0.0876681437449746, 0.0873193439228653, 0.0557886563932414, 0.0697405296684614, 0.0816017043731314, 0.080682483680631, 0.140143018506738, 0.143638554718342, 0.181055291649198, 0.156028064451934, 0.0489887079748926, 0.0568105684499441, 0.0553858354841772, 0.0357634676117837, 0.0632745046660565, 0.0727577335779841, 0.0882028572013856, 0.0873974003528847, 0.0126613951927586, 0.0147981077558741, 0.0159530550200578, 0.00687923775445006, 0.0121335890941154, 0.000966608903165878, 0.000797769801870971, 0.00206496792554405, 0.000751828542342208, 0.000965556138741714, -0.00209496583613917, -0.00829883744654751, -0.00156139084505394, -0.000951312058764824, -0.00864565589354287, 0.000878549600895942, 0.00177686186199479, -0.00512263177659919, -0.00495041484077616, -0.000146088846912167,

0.0608445552931613, 0.078208587237125, 0.104002214972856, 0.128066936456592, 0.0448550876486579, 0.0549410190376934, 0.0647805241463607, 0.0685351372233606, 0.141303324531503, 0.134285751709489, 0.156028064451926, 0.215936294130601, 0.0362119471338291, 0.0382842464212975, 0.039907998311495, 0.0382434907838302, 0.0587615471115301, 0.067330598230502, 0.0851235254157852, 0.100931375739162, 0.0184981709968543, 0.014244122725636, 0.0265682292489127, 0.00638334496104276, 0.014422987425447, 0.000879236234975349, 0.00060551296499323, 0.00176454349936178, 0.000538242693321678, 0.000892523591985909, -0.00469642314806256, -0.00712356004254439, -0.00450349379268551, -0.00292685421483959, -0.00630058553646661, -0.00229626105423547, 0.000257399818882582, -0.00440292593788135, -0.00397859976538834, -0.00171621300391558,

0.0540288483709648, 0.063258971116666, 0.0741039338661626, 0.0654923164116947, 0.068612723995637, 0.0932041929547122, 0.0979268907730152, 0.113520959160058, 0.0452774767219661, 0.0502154371589509, 0.0489887079749024, 0.0362119471338382, 0.173486138091295, 0.216747884568665, 0.221427068703732, 0.257357480103999, 0.0779406675250212, 0.0962480813064225, 0.100831093422004, 0.108073048095595, 0.0103261515585211, 0.0213494842238005, 0.0047295525763676, 0.0502767462607263, 0.0226105921985083, 0.000756565473080054, 0.000930480978005784, 0.000710396873751179, 0.00176220016502761, 0.000963983557759939, -0.00818414379432465, -0.00181654809234533, -0.00840694675062064, -0.00565776834885481, -0.00460924019071226, -0.00313984153717018, -0.00444274662754014, 0.00210433904096146, -0.00392706018901409, -0.00406045284412083,

0.0619828922843558, 0.0800186692796267, 0.0967259878871404, 0.0862799317418954, 0.0684145376240138, 0.102102008516123, 0.112180954038331, 0.131729771972816, 0.047040597451591, 0.0499121116119968, 0.0568105684499567, 0.0382842464213066, 0.216747884568664, 0.324071209787444, 0.320860711722874, 0.369201596131723, 0.0858231926920983, 0.112358857709566, 0.129052105131397, 0.143038107190847, 0.00988754184009695, 0.0278184652235355, 0.00547330774727167, 0.0721106269867835, 0.0319300603939522, 0.000987533490481229, 0.000907028410446433, 0.000705014917805112, 0.0023750600982776, 0.00106322252260454, -0.00963087563266194, -0.00188275892459553, -0.010222137493449, -0.00743102393686105, -0.00511634980077334, -0.00666227734079867, -0.00609747661316802, 0.0055163336226731, -0.0052960288591643, -0.00549487477918098,

0.0631140319474603, 0.0813112087210689, 0.105766875721289, 0.09583898004192, 0.0761415619958523, 0.113766168130699, 0.134175921025067, 0.164771190917237, 0.0435535345797671, 0.0499550469062345, 0.0553858354841913, 0.039907998311507, 0.221427068703733, 0.320860711722876, 0.355243981466611, 0.397090854899178, 0.0900361679313647, 0.119577376265341, 0.149250765706892, 0.156493139486865, 0.012367594181835, 0.0341124446885127, 0.00689888149867242, 0.0723448321862962, 0.0331508045999576, 0.000996067408198294, 0.000990181531497154, 0.000668292413593005, 0.00252156348164975, 0.00118096832244846, -0.0142210784075459, -0.00209197040444141, -0.0125427162735922, -0.0093766923760477, -0.00749737114344219, -0.00939099144183172, -0.00802899398755301, 0.00521364914319603, -0.00502248736731815, -0.00703983623045437,

0.0587727790195394, 0.075345125424576, 0.12006494421048, 0.107689293405187, 0.08523545847321, 0.130202024444085, 0.155289214042845, 0.234101479853763, 0.0270873529989314, 0.0324719667340631, 0.0357634676118036, 0.038243490783851, 0.257357480104006, 0.369201596131734, 0.397090854899187, 0.575887370946566, 0.0718599456027547, 0.0968392480759508, 0.119043780875255, 0.175278185378392, 0.0171869448463073, 0.0457834155107275, 0.00891613798887409, 0.105605742864088, 0.0377572379159157, 0.000806726572223422, 0.000970680941704678, 0.000351288929047825, 0.00238180941843746, 0.000741710759936766, -0.0168029193639947, 0.00100042880250419, -0.0175643176252796, -0.0112541600058259, -0.00800060376281665, -0.0117217569549262, -0.0110821011824901, 0.00831274176684205, -0.00535213119120329, -0.0118404238658249,

0.0555658336990009, 0.0622125231673228, 0.0626301178004865, 0.060798965012652, 0.0712318797122779, 0.0824671224596387, 0.087670107679933, 0.0885724473629524, 0.0618401472590861, 0.0603817713220297, 0.0632745046660596, 0.0587615471115362, 0.0779406675250201, 0.0858231926920967, 0.0900361679313619, 0.0718599456027469, 0.0974731486349393, 0.105902140919425, 0.118855533785074, 0.109728190004613, 0.00803175424309481, 0.0148487302368954, 0.00603175543007465, 0.0141006074697581, 0.0205020993414526, 0.000830456756747556, 0.00100902024792518, 0.000929202996083081, 0.0010381275751292, 0.0012471091968335, -0.00396093348866936, -0.00191720888147532, -0.00424904522778932, -0.00184466162304437, -0.00184011447017086, -0.00302028190273773, -0.000853503894470119, -0.00168936170790527, -8.7193611382396e-05, 0.000121941905861159,

0.0614511294772137, 0.0761296075375762, 0.0786805763223996, 0.0772454576219088, 0.0762040877550106, 0.101367504732492, 0.110732590748989, 0.111509638952134, 0.0651968913342681, 0.0697953748435198, 0.0727577335779885, 0.0673305982305097, 0.0962480813064207, 0.112358857709564, 0.119577376265338, 0.0968392480759415, 0.105902140919425, 0.143155649805257, 0.154928836953842, 0.141502361256166, 0.00941442990173387, 0.0173870659347696, 0.00512151271966227, 0.0180890856695447, 0.0246350449660914, 0.000981049597574479, 0.0011957371793211, 0.00106563907237629, 0.00127903124246732, 0.00153875188603475, -0.00847236667992239, -0.00522116705604771, -0.00683577726650223, -0.00377466510201385, -0.00441839752518169, -0.0061434370069086, -0.00252828945955109, -0.00426963879823508, -0.001109078933399, -0.00103533522200497,

0.064154848877662, 0.07882674753533, 0.101112507536065, 0.100172386692143, 0.0786040582730614, 0.106966338018308, 0.131039484851323, 0.135616283976293, 0.0660902265535449, 0.0721582859464907, 0.0882028572013898, 0.0851235254157908, 0.100831093422005, 0.129052105131397, 0.149250765706891, 0.119043780875246, 0.118855533785074, 0.154928836953842, 0.218336911080434, 0.188142887437716, 0.0163515314225692, 0.0241422884233445, 0.00733620763271582, 0.022168397804343, 0.0370106529609202, 0.000943476862706621, 0.00116946822965691, 0.00111424617445052, 0.00139019505718905, 0.00164671781075745, -0.0149715766924478, -0.00639437993064049, -0.0108049945748763, -0.00511524713847237, -0.00437844996456449, -0.00991172509577811, -0.00474275699588615, -0.00473901902811135, -0.00186195485850043, -0.00334786725395455,

0.061288187970073, 0.0767731449022485, 0.101362605963156, 0.110455385528046, 0.0827520444382618, 0.113028470156395, 0.139391078896377, 0.170490968111407, 0.0663800061981904, 0.0741303629456559, 0.0873974003528888, 0.100931375739168, 0.108073048095596, 0.14303810719085, 0.156493139486866, 0.175278185378386, 0.109728190004613, 0.141502361256166, 0.188142887437717, 0.201977729531031, 0.0190068675573827, 0.0315692674577327, 0.0116226279703413, 0.031798599227366, 0.0392488476681211, 0.000874374175024732, 0.00115444887117555, 0.00107095177094962, 0.00133017906966978, 0.00144981201598893, -0.0148237613326105, -0.00591401054408084, -0.014055164018471, -0.00577719412173489, -0.00651742872795177, -0.011058076723614, -0.00548936241879543, -0.00519228894051092, -0.00320519114046342, -0.00463030066436005,

0.0112006963283242, 0.0114242086825808, 0.0259908881385258, 0.026711155330212, 0.00763426281828802, 0.0108154213009743, 0.0167052729480279, 0.0242979064656269, 0.00813233281744219, 0.00953578499131538, 0.0126613951927605, 0.0184981709968564, 0.0103261515585198, 0.00988754184009525, 0.0123675941818331, 0.0171869448463045, 0.00803175424309457, 0.00941442990173374, 0.0163515314225689, 0.0190068675573824, 0.0269744461481307, 0.018714148970998, 0.0161909943008128, 0.0173070707548299, 0.0196154401925865, -0.000368158374411348, -0.000200978893329087, -0.000187827205966309, -0.000228369672774404, -0.000249615452084476, -0.0021953302465838, -0.00133223622741572, -0.00222565439001593, -0.000895974454377887, -0.00122558402552068, -0.000625419824641894, -0.000311364386470513, -0.000368160785668035, 0.000136522198247627, -0.00125868249605014,

0.00961279618616583, 0.0137893469782209, 0.0222938607875486, 0.0233556272960906, 0.0231314472853493, 0.0299971924720924, 0.043849076697349, 0.0679713970039115, 0.00617348485207109, 0.0112337464420093, 0.0147981077558768, 0.0142441227256382, 0.0213494842238023, 0.0278184652235385, 0.0341124446885156, 0.0457834155107301, 0.0148487302368957, 0.0173870659347701, 0.0241422884233447, 0.031569267457733, 0.018714148970999, 0.0353105007998933, 0.0127635390858482, 0.0249775695280664, 0.0238106244294416, -0.000163981454614356, -0.000222461737275698, -9.38159078190193e-05, -0.000149122591782057, -0.000202772563107446, -0.00351579169379637, -0.00120631722590624, -0.00302181189255591, -0.000998675337061257, -0.00274148716872987, -0.00243634269713134, -0.00181972652702873, -0.000159900881097013, 0.000958766880180706, -0.00207209830406477,

0.00782461531119497, 0.00934883549715236, 0.0132034058657455, 0.015493072555828, 0.00607423149725652, 0.00553500598291318, 0.00672250388217795, 0.0113794484971461, 0.01697174798979, 0.0153174042247213, 0.0159530550200576, 0.0265682292489139, 0.004729552576367, 0.00547330774727141, 0.00689888149867188, 0.00891613798887324, 0.00603175543007419, 0.00512151271966179, 0.00733620763271568, 0.0116226279703414, 0.0161909943008128, 0.0127635390858477, 0.0360923561651482, 0.0136442667428943, 0.0126427295715375, -0.000219471961840495, -0.000168265680005513, -0.000597722804944346, -0.000212803669705602, -0.00014699152151314, -0.00318626039585589, 0.000266133313947107, 0.000109634880712406, -0.000219629434327145, 0.000126398003277893, -0.000408689085179132, 0.000480355173848312, -0.00051804145377093, -0.000913898302957811, -0.000377693366775495,

0.00921422224757468, 0.0105552827567451, 0.0180595074953376, 0.0174849361925336, 0.0130228439039837, 0.021377601231737, 0.0285859844152996, 0.037963205956935, 0.00130705755598347, 0.00363334587089299, 0.00687923775445322, 0.00638334496104569, 0.0502767462607271, 0.0721106269867851, 0.0723448321862968, 0.105605742864086, 0.0141006074697585, 0.0180890856695454, 0.0221683978043434, 0.0317985992273662, 0.0173070707548306, 0.0249775695280656, 0.0136442667428945, 0.0498924191175863, 0.0269722740481501, -0.000227458652421661, -0.000242425069947961, -0.00023582493438877, -0.000286670102405827, -0.000327881564198946, -0.00343805843880051, 0.000336404341545734, -0.00266356708534915, -0.00298444497129765, -0.00194563953701841, -0.00227055257338718, -0.00193113701677775, 0.00128692172913462, -0.00249423304806358, -0.00197053191940498,

0.00837842914724268, 0.0134736696483176, 0.0198696261139965, 0.0209492699125723, 0.0142562747908322, 0.0203063142886042, 0.0251502126844574, 0.0326933593555022, 0.00967825339587032, 0.0101969201783201, 0.0121335890941176, 0.0144229874254497, 0.0226105921985083, 0.0319300603939522, 0.0331508045999573, 0.0377572379159143, 0.0205020993414529, 0.0246350449660917, 0.0370106529609203, 0.0392488476681215, 0.0196154401925867, 0.0238106244294411, 0.0126427295715375, 0.0269722740481499, 0.0344539751120323, -0.000224007328365485, -0.000194112041701624, -6.39342004303372e-05, -0.000175357817960948, -0.000347612428965594, -0.0024589550309865, -0.000599256477605256, -0.00452728376162534, -0.00123555593446847, -0.00112283401755342, -0.00175144220881373, -0.000840600805172513, -0.000135408885841132, -0.000323759147266236, -0.0011209890065146,

0.00112663340003969, 0.00151668466288831, 0.0014338192445327, 0.00118727103016332, 0.000682446551014051, 0.000703612983918196, 0.000731730760982449, 0.000605746874659924, 0.000961224342272526, 0.00094420839944873, 0.000966608903165933, 0.00087923623497545, 0.000756565473079914, 0.000987533490481034, 0.000996067408198087, 0.000806726572223136, 0.000830456756747536, 0.000981049597574448, 0.000943476862706598, 0.000874374175024696, -0.000368158374411348, -0.000163981454614352, -0.000219471961840477, -0.000227458652421697, -0.00022400732836549, 3.39506864636966e-05, 1.78473528405874e-05, 2.41335201933233e-05, 2.29455281649813e-05, 2.30356445382745e-05, -1.18274259561623e-05, -2.01531971601169e-05, 2.65880925232215e-05, -2.795948115949e-05, -9.88960765830572e-06, -2.48975083067052e-05, -3.46382610940521e-07, -1.11527343679843e-05, -9.70090469987749e-06, 1.00935952461452e-05,

0.00064075218581095, 0.00066868682433253, 0.000583887643970163, 0.00055778951408584, 0.0013382766822423, 0.00164545291942405, 0.00170942597632534, 0.00187300878697362, 0.000754678784243217, 0.000783984843068038, 0.00079776980187105, 0.000605512964993156, 0.000930480978005899, 0.000907028410446574, 0.000990181531497289, 0.000970680941704784, 0.00100902024792522, 0.00119573717932116, 0.00116946822965694, 0.00115444887117553, -0.000200978893329066, -0.000222461737275661, -0.000168265680005498, -0.000242425069947919, -0.000194112041701602, 1.78473528405896e-05, 3.4663145348525e-05, 1.86145178918137e-05, 2.46517474793249e-05, 2.64011603806216e-05, -2.09522241463196e-05, 9.85218950659092e-06, -0.000123937434459911, -1.22575021303379e-05, -2.67701164341923e-05, -2.7332320293133e-05, -2.86251933664195e-05, -1.55596508466911e-05, -1.12978508399906e-07, -2.61977124216585e-05,

0.000838141109400295, 0.00100094517829637, 0.00100009201059669, 0.000997419267902765, 0.000715172727819466, 0.000842953633599351, 0.000897488895155287, 0.000832115075863021, 0.00206925424816071, 0.00186932725795986, 0.002064967925544, 0.00176454349936174, 0.000710396873751036, 0.000705014917804913, 0.00066829241359278, 0.00035128892904752, 0.000929202996083005, 0.00106563907237619, 0.00111424617445039, 0.00107095177094947, -0.000187827205966348, -9.38159078190503e-05, -0.000597722804944353, -0.000235824934388817, -6.39342004303741e-05, 2.41335201933222e-05, 1.86145178918129e-05, 5.08123772819197e-05, 2.10417882508273e-05, 2.19594474387598e-05, 2.75263173629528e-05, -8.31601131782329e-05, -9.02076089310113e-05, -3.06017625533023e-05, -8.60767156116869e-05, -5.52574824033225e-06, -3.66984175011784e-06, -3.09286036874987e-05, -2.26839130588266e-05, 1.05369684715933e-05,

0.000742435524876919, 0.000894722909358352, 0.00100186238932461, 0.000857741991673102, 0.000893115634793061, 0.00109634007814385, 0.00106803539330138, 0.00120822934274978, 0.00091027972652031, 0.000828205555254935, 0.000751828542342315, 0.000538242693321784, 0.00176220016502761, 0.00237506009827759, 0.00252156348164974, 0.00238180941843736, 0.00103812757512923, 0.00127903124246734, 0.00139019505718905, 0.00133017906966977, -0.000228369672774401, -0.000149122591782076, -0.000212803669705598, -0.000286670102405843, -0.000175357817960955, 2.29455281649831e-05, 2.46517474793242e-05, 2.10417882508291e-05, 4.53680352969705e-05, 2.8869239150439e-05, -4.18819668893153e-05, -3.3164795111976e-05, -0.000103871462550543, -2.12339177912497e-05, -4.019960052227e-05, -3.60729052131166e-05, -3.49769165428667e-05, 4.89565517813121e-05, 1.08509563770283e-05, -3.09819549819341e-05,

0.000793761360187231, 0.000815885984607273, 0.00081114186922162, 0.000801909567185656, 0.000965188314493341, 0.00111541811646086, 0.00118251237624348, 0.00113432287578025, 0.00100868166187509, 0.000939682926880678, 0.000965556138741724, 0.000892523591985991, 0.000963983557759925, 0.00106322252260452, 0.00118096832244843, 0.000741710759936651, 0.00124710919683349, 0.00153875188603473, 0.00164671781075745, 0.00144981201598893, -0.000249615452084483, -0.00020277256310745, -0.000146991521513136, -0.000327881564198953, -0.000347612428965609, 2.30356445382747e-05, 2.64011603806217e-05, 2.19594474387609e-05, 2.88692391504386e-05, 3.62269874182728e-05, -6.40960017232696e-05, -4.36363284202697e-05, -3.17743195792508e-05, -1.97654434435282e-05, -2.52948849914584e-05, -5.92230002473967e-05, -2.35246327360209e-05, -2.54223558305405e-05, 5.75331275670525e-06, 7.01234461791427e-06,

-0.00133930573499522, -0.0029848150634285, -0.00771449830405123, -0.00748741357277635, -0.00208065478519311, -0.00615899283341022, -0.0104195078240247, -0.0148285766963694, 0.00130251077532943, -0.00282121235614817, -0.00209496583613971, -0.00469642314806229, -0.00818414379432575, -0.00963087563266351, -0.0142210784075475, -0.0168029193639962, -0.00396093348866963, -0.00847236667992282, -0.014971576692448, -0.0148237613326106, -0.00219533024658387, -0.00351579169379643, -0.00318626039585592, -0.00343805843880084, -0.00245895503098664, -1.18274259561675e-05, -2.0952224146324e-05, 2.75263173629447e-05, -4.18819668893266e-05, -6.40960017232696e-05, 0.00769071691768088, 0.00159123262572133, 0.00177603715540889, 0.00172382191080989, 0.000974813527374095, 0.00245232472925846, 0.00140628192413722, 0.00170009543230041, 0.00108738495945332, 0.00088779816025713,

-0.000497848909223235, -0.00379229437967505, -0.0054132233490578, -0.00549826852062474, -0.000673363649258823, -0.000918898327170961, -0.00146003878922384, -0.000879486994537901, -0.000541963706084296, -0.00554113859624245, -0.00829883744654807, -0.00712356004254511, -0.00181654809234512, -0.0018827589245952, -0.00209197040444115, 0.001000428802505, -0.00191720888147542, -0.00522116705604788, -0.00639437993064047, -0.00591401054408092, -0.00133223622741566, -0.00120631722590611, 0.000266133313947085, 0.000336404341545894, -0.000599256477605211, -2.01531971601188e-05, 9.85218950659085e-06, -8.316011317824e-05, -3.31647951119764e-05, -4.36363284202747e-05, 0.00159123262572132, 0.00272821149006434, 0.000175699173713124, 0.000518460523991481, 0.00159519508667298, 0.000501389596675838, 3.17339994681569e-05, 0.00190326704409882, 0.00158055496779657, 0.000303164240016541,

0.000789816506243392, -0.000153892962075076, -0.0082342636971114, -0.00727935962738955, -0.00657020355986639, -0.0107220997568328, -0.0127638582988782, -0.0231374327825337, -0.00530097743908021, -0.00424292917769086, -0.0015613908450549, -0.0045034937926864, -0.00840694675062172, -0.0102221374934504, -0.0125427162735939, -0.017564317625281, -0.00424904522778983, -0.00683577726650287, -0.0108049945748768, -0.0140551640184715, -0.00222565439001596, -0.00302181189255585, 0.000109634880712504, -0.00266356708534943, -0.0045272837616254, 2.65880925232082e-05, -0.000123937434459919, -9.02076089310301e-05, -0.000103871462550553, -3.1774319579258e-05, 0.00177603715540889, 0.00017569917371313, 0.00680202480218658, 0.00169689284851416, 0.000843292570658534, 0.00133459366842304, 0.000904573520309146, 0.000671765948712495, 0.000517623560785999, 0.00167059983295353,

-0.00118563096723427, -0.00436134943949042, -0.00539218716136602, -0.00561515174896733, -0.000352332962434616, -0.00193299666211695, -0.00354902449956855, -0.00531231659307419, -0.000128799873373622, -0.00297879005007047, -0.000951312058765451, -0.00292685421484015, -0.00565776834885466, -0.00743102393686087, -0.00937669237604757, -0.0112541600058258, -0.00184466162304455, -0.00377466510201409, -0.00511524713847258, -0.00577719412173507, -0.000895974454377896, -0.00099867533706127, -0.000219629434327105, -0.00298444497129761, -0.00123555593446853, -2.79594811594939e-05, -1.22575021303324e-05, -3.0601762553314e-05, -2.12339177912503e-05, -1.97654434435324e-05, 0.00172382191080987, 0.000518460523991502, 0.00169689284851415, 0.00255173561109025, 0.000837531336670912, 0.000593037367701464, 0.000546433143651793, 0.000826831674336257, 0.00158508850051193, 0.000593922518782946,

-0.00207735259778344, -0.00481974992215261, -0.00381782689489326, -0.00505491061038999, -0.000394509568453431, -0.00599550169747857, -0.0077626925722646, -0.00998871836530529, 0.00207249105543952, -0.00774324488350188, -0.00864565589354305, -0.00630058553646684, -0.00460924019071173, -0.00511634980077289, -0.00749737114344166, -0.00800060376281616, -0.0018401144701706, -0.00441839752518144, -0.00437844996456415, -0.00651742872795141, -0.00122558402552058, -0.00274148716872971, 0.000126398003277888, -0.00194563953701828, -0.0011228340175533, -9.88960765830033e-06, -2.6770116434184e-05, -8.6076715611691e-05, -4.01996005222671e-05, -2.52948849914594e-05, 0.000974813527374048, 0.00159519508667297, 0.000843292570658475, 0.000837531336670873, 0.00462123952436938, 0.000461666132928993, 0.000262303116942818, 0.00310897878289472, 0.00378409150946364, 0.000641773174391947,

-0.00111751680135707, -0.000375511394176536, -0.00434435112911292, -0.00632141927926294, -0.00230687185109581, -0.00485499030221217, -0.00945089478487532, -0.0124469157575272, 0.0010162382763995, -0.000167531084234554, 0.000878549600896046, -0.00229626105423537, -0.00313984153717103, -0.00666227734080006, -0.00939099144183294, -0.011721756954927, -0.00302028190273782, -0.00614343700690868, -0.00991172509577851, -0.0110580767236142, -0.000625419824641976, -0.00243634269713148, -0.000408689085179112, -0.00227055257338743, -0.00175144220881377, -2.48975083067076e-05, -2.73323202931513e-05, -5.52574824034251e-06, -3.60729052131254e-05, -5.9223000247394e-05, 0.00245232472925856, 0.000501389596675831, 0.00133459366842303, 0.000593037367701468, 0.000461666132929003, 0.00263453347686425, 0.00129192391940605, 0.000794793756903752, 0.000428926519353624, 0.00088514393551457,

0.000149852559388207, 0.00131211337140294, -0.00173225984370912, -0.00239524619341665, -0.00225379706937111, -0.00505054844487333, -0.00751525518607402, -0.00993965956814431, 0.00181663003374032, 0.000663795942712322, 0.00177686186199466, 0.000257399818882617, -0.00444274662754107, -0.00609747661316943, -0.00802899398755433, -0.0110821011824912, -0.000853503894470281, -0.0025282894595513, -0.00474275699588645, -0.00548936241879567, -0.0003113643864706, -0.00181972652702883, 0.000480355173848314, -0.00193113701677802, -0.000840600805172571, -3.46382610945422e-07, -2.86251933664305e-05, -3.6698417501246e-06, -3.49769165428769e-05, -2.35246327360209e-05, 0.00140628192413726, 3.17339994681646e-05, 0.000904573520309157, 0.000546433143651808, 0.000262303116942831, 0.00129192391940604, 0.00110223031504778, 0.000154801243811681, 0.000256207478751857, 0.000574918310734515,

-0.00149219667391203, -0.00296326635195771, -0.00235439262059322, -0.00317135815187522, -0.000639983059221946, -0.00140568607572505, -0.00252224039965014, -0.00365997145496467, 0.00638045187237137, -0.00456032716790112, -0.00512263177659848, -0.00440292593788069, 0.00210433904096211, 0.00551633362267386, 0.00521364914319683, 0.00831274176684277, -0.00168936170790476, -0.0042696387982345, -0.00473901902811075, -0.00519228894051034, -0.000368160785667824, -0.00015990088109699, -0.000518041453770886, 0.00128692172913477, -0.000135408885840979, -1.11527343679693e-05, -1.55596508466901e-05, -3.09286036874911e-05, 4.89565517813201e-05, -2.54223558305344e-05, 0.00170009543230042, 0.00190326704409878, 0.000671765948712474, 0.000826831674336219, 0.00310897878289471, 0.000794793756903757, 0.000154801243811701, 0.00453853476811452, 0.00459351988596583, 0.00054245984442212,

-0.00113488560297151, -0.00292991341196714, -0.00255151662082212, -0.00254708842935712, 0.00100817289209192, 0.00130107434513197, 0.000550359773864661, 0.00135998034527161, 0.00615250158217959, -0.00414611327679648, -0.00495041484077573, -0.00397859976538776, -0.0039270601890135, -0.0052960288591635, -0.0050224873673173, -0.0053521311912024, -8.71936113819812e-05, -0.00110907893339851, -0.00186195485849987, -0.00320519114046278, 0.000136522198247788, 0.000958766880180828, -0.000913898302957743, -0.00249423304806338, -0.000323759147266106, -9.7009046998698e-06, -1.12978508394389e-07, -2.26839130588214e-05, 1.08509563770351e-05, 5.7533127567108e-06, 0.00108738495945329, 0.00158055496779655, 0.000517623560785961, 0.00158508850051191, 0.00378409150946362, 0.000428926519353588, 0.000256207478751839, 0.00459351988596584, 0.00688929575519049, 0.000430601378397385,

-0.00020027821398438, -4.43077964770517e-05, -0.00334040119800445, -0.00406856559644027, -0.00222457604061856, -0.00432850981824581, -0.00608511348814453, -0.0115706757465204, 0.00174512170572537, -0.000416742948961501, -0.000146088846912583, -0.00171621300391611, -0.00406045284412144, -0.00549487477918194, -0.00703983623045525, -0.0118404238658256, 0.000121941905860851, -0.00103533522200535, -0.00334786725395499, -0.00463030066436042, -0.00125868249605022, -0.00207209830406476, -0.000377693366775495, -0.00197053191940509, -0.00112098900651465, 1.00935952461359e-05, -2.61977124216661e-05, 1.05369684715826e-05, -3.09819549819426e-05, 7.01234461791077e-06, 0.000887798160257153, 0.000303164240016572, 0.00167059983295353, 0.000593922518782962, 0.000641773174391959, 0.000885143935514565, 0.000574918310734504, 0.000542459844422128, 0.000430601378397413, 0.00139770460050363),

nrow=40,ncol=40,byrow = TRUE)

# next compute the Cholesky decomposition

chol_1b <- chol(var_cov_1b)

# transpose the Cholesky decomposition

chol_1b <- t(chol_1b)

# produce a random draw for each parameter

random.draw <- rnorm(40,0,1)

# this information can now be combined with the cholesky decomposition

# the Tz terms reflect sample deviations away from the parameter point estimates

Tz <- NULL

for(i in 1:40){

Tz <- rbind(Tz,random.draw%*%chol_1b[i,])

}

# applying Tz terms to the error structure

if(probabilistic==1)

sigma <- matrix(

data = c(1.0000000,0.6437712+Tz[31],0.7912376+Tz[32],0.7703542+Tz[33],

0.8283033+Tz[34],0.6437712+Tz[31],1.0000000,

0.6627683+Tz[35],0.8595744+Tz[36],0.905238+Tz[37],

0.7912376+Tz[32],0.6627683+Tz[35],1.0000000,

0.6439538+Tz[38],0.5946516+Tz[39],0.7703542+Tz[33],

0.8595744+Tz[36],0.6439538+Tz[38],1.0000000,

0.8944501+Tz[40],0.8283033+Tz[34],0.905238+Tz[37],

0.5946516+Tz[39],0.8944501+Tz[40],1.0000000),

nrow = 5, ncol = 5, byrow = TRUE,

dimnames = list(c("ad","mo","pd","sc","ua"),

c("ad","mo","pd","sc","ua")))

# applying Tz terms to the regression coefficients

if(probabilistic==1)

coefficients <- c(0.73055820+Tz[21], 0.97200502+Tz[22], 0.61191017+Tz[23],

1.08819221+Tz[24], 0.96146978+Tz[25],0.01786853+Tz[26],

0.01498625+Tz[27], 0.02299354+Tz[28], 0.02186020+Tz[29],

0.01381784+Tz[30])

# applying Tz terms to the threshold values

if(probabilistic==1)

theta <- list(c(1.597304+Tz[1], 2.606510+Tz[2], 3.701212+Tz[3], 4.289984+Tz[4]),

c(1.115285+Tz[5], 2.759396+Tz[6], 3.462357+Tz[7], 4.500355+Tz[8]),

c(-0.6394355+Tz[9], 2.3960214+Tz[10], 3.2310568+Tz[11], 4.3407331+Tz[12]),

c(2.895494+Tz[13], 3.837782+Tz[14], 4.395331+Tz[15], 5.321474+Tz[16]),

c(1.781658+Tz[17], 2.675991+Tz[18], 3.528780+Tz[19], 4.133645+Tz[20]))

################################################################################

### Stage 4 (Model 1b): code below based on get.prob() function (see mvord package)

### this calculates the probability of different health

### state responses occurring

################################################################################

S_full <- c("1","1","1","1","1")

S <- list(

list(c("2",NA,NA,NA,NA),c("3",NA,NA,NA,NA),c("4",NA,NA,NA,NA),c("5",NA,NA,NA,NA)),

list(c(NA,"2",NA,NA,NA),c(NA,"3",NA,NA,NA),c(NA,"4",NA,NA,NA),c(NA,"5",NA,NA,NA)),

list(c(NA,NA,"2",NA,NA),c(NA,NA,"3",NA,NA),c(NA,NA,"4",NA,NA),c(NA,NA,"5",NA,NA)),

list(c(NA,NA,NA,"2",NA),c(NA,NA,NA,"3",NA),c(NA,NA,NA,"4",NA),c(NA,NA,NA,"5",NA)),

list(c(NA,NA,NA,NA,"2"),c(NA,NA,NA,NA,"3"),c(NA,NA,NA,NA,"4"),c(NA,NA,NA,NA,"5")))

for(k in 1:5){

names(S[[k]]) <- c("L2","L3","L4","L5")

}

levels <- list(c("1","2","3","4","5"),

c("1","2","3","4","5"),

c("1","2","3","4","5"),

c("1","2","3","4","5"),

c("1","2","3","4","5"))

n <- length(predictors[,1])

subjectID <- c(1:n)

if (is.null(subjectID))

ind <- c(1:n)

if(any(complete.cases(subjectID)))

ind <- subjectID

stddevs <- sqrt(diag(sigma))

constraints <- matrix(

data = c(rep(c(1,0,0,0,0),4),

rep(c(0,1,0,0,0),4),

rep(c(0,0,1,0,0),4),

rep(c(0,0,0,1,0),4),

rep(c(0,0,0,0,1),4)),

nrow = 20, ncol = 5, byrow = TRUE)

inds.cat <- list(c(1,2,3,4),

c(5,6,7,8),

c(9,10,11,12),

c(13,14,15,16),

c(17,18,19,20))

select <- list(c(1:5),c(6:10))

full_health <- 1

full_health[is.null(S_full)] <- 0

elements <- full_health + length(S[[1]]) + length(S[[2]]) + length(S[[3]]) +

length(S[[4]]) + length(S[[5]])

probabilities <- NULL

state <- NULL

for(l in 1:elements){

response.cat <- NULL

if(l==1&!is.null(S_full))

response.cat <- S_full

if(l==1&is.null(S_full))

response.cat <- S[[1]][[1]]

list_cat_1 <- 1

list_cat_1[(l-1)>length(S[[1]])] <- 2

list_cat_1[(l-1)>(length(S[[1]])+length(S[[2]]))] <- 3

list_cat_1[(l-1)>(length(S[[1]])+length(S[[2]])+length(S[[3]]))] <- 4

list_cat_1[(l-1)>(length(S[[1]])+length(S[[2]])+length(S[[3]])+length(S[[4]]))] <- 5

list_cat_2 <- l - 1

list_cat_2[list_cat_1==2] <- l - 1 - length(S[[1]])

list_cat_2[list_cat_1==3] <- l - 1 - length(S[[1]]) - length(S[[2]])

list_cat_2[list_cat_1==4] <- l - 1 - length(S[[1]]) - length(S[[2]]) - length(S[[3]])

list_cat_2[list_cat_1==5] <- l - 1 - length(S[[1]]) - length(S[[2]]) - length(S[[3]]) -

length(S[[4]])

if(l>=2&!is.null(S_full))

response.cat <- S[[list_cat_1]][[list_cat_2]]

list_cat_1 <- 1

list_cat_1[l>length(S[[1]])] <- 2

list_cat_1[l>(length(S[[1]])+length(S[[2]]))] <- 3

list_cat_1[l>(length(S[[1]])+length(S[[2]])+length(S[[3]]))] <- 4

list_cat_1[l>(length(S[[1]])+length(S[[2]])+length(S[[3]])+length(S[[4]]))] <- 5

list_cat_2 <- l

list_cat_2[list_cat_1==2] <- l - length(S[[1]])

list_cat_2[list_cat_1==3] <- l - length(S[[1]]) - length(S[[2]])

list_cat_2[list_cat_1==4] <- l - length(S[[1]]) - length(S[[2]]) - length(S[[3]])

list_cat_2[list_cat_1==5] <- l - length(S[[1]]) - length(S[[2]]) - length(S[[3]]) -

length(S[[4]])

if(l>=2&is.null(S_full))

response.cat <- S[[list_cat_1]][[list_cat_2]]

state <- rbind(state,response.cat)

if (is.vector(response.cat))

response.cat <- matrix(response.cat, ncol = length(response.cat),

nrow = length(ind), byrow = TRUE)

response.cat <- lapply(1:5, function(j) {

ordered(response.cat[, j], levels = levels[[j]])

})

# 5

XcatL <- list()

XcatU <- list()

for (j in 1:5) {

ncat <- 4

mm <- model.matrix(~-1 +

response.cat[[j]]:predictors[ind,],

model.frame(~-1 + response.cat[[j]]:predictors[ind,],

na.action = function(x) x))

XcatL[[j]] <- mm[, -(ncat * (seq_len(ncol(predictors)) - 1) + 1), drop = F]

XcatU[[j]] <- mm[, -(ncat * seq_len(ncol(predictors))), drop = F]

}

# 6

pred.fixedU <- sapply(1:5, function(j) {

b <- lapply(seq_len(ncol(predictors)),

function(k) constraints[inds.cat[[j]],

, drop = F] %*% coefficients[select[[k]]])

XcatU[[j]] %*% unlist(b)

})

pred.fixedL <- sapply(1:5, function(j) {

b <- lapply(seq_len(ncol(predictors)),

function(i) constraints[inds.cat[[j]],

, drop = F] %*% coefficients[select[[i]]])

XcatL[[j]] %*% unlist(b)

})

# 7

if (is.null(dim(pred.fixedU)))

dim(pred.fixedU) <- c(1, length(pred.fixedU))

if (is.null(dim(pred.fixedL)))

dim(pred.fixedL) <- c(1, length(pred.fixedL))

# 8

theta.lower <- sapply(1:5, function(j) c(-10000,theta[[j]]) [response.cat[[j]]])

theta.upper <- sapply(1:5, function(j) c(theta[[j]],10000)[response.cat[[j]]])

# 9

pred.lower <- (theta.lower - pred.fixedL)/stddevs

pred.upper <- (theta.upper - pred.fixedU)/stddevs

pred.lower[is.na(pred.lower)] <- -10000

pred.upper[is.na(pred.upper)] <- 10000

list_R = cov2cor(sigma)

prob <- sapply(1:nrow(pred.upper), function(i) sadmvn(lower = pred.lower[i,],

upper = pred.upper[i,],

mean = rep(0, NCOL(pred.upper)),

varcov = list_R))

probabilities <- cbind(probabilities,prob)

}

state <- data.frame(state)

probabilities <- data.frame(probabilities)

################################################################################

### Step 5 (Model 1b): the code below calculate the expected EQ-5D values by

### combining the probabilities derived in the previous step

### with the value weights from Luo, Liu et al. (2017)

################################################################################

full_health <- rep(0,length(ind))

if(!is.null(S_full))

full_health <- probabilities[,1]

dec_ad2 <- rep(0,length(ind))

if(any(complete.cases(state[,1])&state[,1]=="2"))

dec_ad2 <- probabilities[,which(state[,1]=="2")]*0.258

dec_ad3 <- rep(0,length(ind))

if(any(complete.cases(state[,1])&state[,1]=="3"))

dec_ad3 <- probabilities[,which(state[,1]=="3")]*0.258

dec_ad4 <- rep(0,length(ind))

if(any(complete.cases(state[,1])&state[,1]=="4"))

dec_ad4 <- probabilities[,which(state[,1]=="4")]*0.258

dec_ad5 <- rep(0,length(ind))

if(any(complete.cases(state[,1])&state[,1]=="5"))

dec_ad5 <- probabilities[,which(state[,1]=="5")]*0.258

dec_mo2 <- rep(0,length(ind))

if(any(complete.cases(state[,2])&state[,2]=="2"))

dec_mo2 <- probabilities[,which(state[,2]=="2")]*0.345

dec_mo3 <- rep(0,length(ind))

if(any(complete.cases(state[,2])&state[,2]=="3"))

dec_mo3 <- probabilities[,which(state[,2]=="3")]*0.345

dec_mo4 <- rep(0,length(ind))

if(any(complete.cases(state[,2])&state[,2]=="4"))

dec_mo4 <- probabilities[,which(state[,2]=="4")]*0.345

dec_mo5 <- rep(0,length(ind))

if(any(complete.cases(state[,2])&state[,2]=="5"))

dec_mo5 <- probabilities[,which(state[,2]=="5")]*0.345

dec_pd2 <- rep(0,length(ind))

if(any(complete.cases(state[,3])&state[,3]=="2"))

dec_pd2 <- probabilities[,which(state[,3]=="2")]*0.302

dec_pd3 <- rep(0,length(ind))

if(any(complete.cases(state[,3])&state[,3]=="3"))

dec_pd3 <- probabilities[,which(state[,3]=="3")]*0.302

dec_pd4 <- rep(0,length(ind))

if(any(complete.cases(state[,3])&state[,3]=="4"))

dec_pd4 <- probabilities[,which(state[,3]=="4")]*0.302

dec_pd5 <- rep(0,length(ind))

if(any(complete.cases(state[,3])&state[,3]=="5"))

dec_pd5 <- probabilities[,which(state[,3]=="5")]*0.302

dec_sc2 <- rep(0,length(ind))

if(any(complete.cases(state[,4])&state[,4]=="2"))

dec_sc2 <- probabilities[,which(state[,4]=="2")]*0.253

dec_sc3 <- rep(0,length(ind))

if(any(complete.cases(state[,4])&state[,4]=="3"))

dec_sc3 <- probabilities[,which(state[,4]=="3")]*0.253

dec_sc4 <- rep(0,length(ind))

if(any(complete.cases(state[,4])&state[,4]=="4"))

dec_sc4 <- probabilities[,which(state[,4]=="4")]*0.253

dec_sc5 <- rep(0,length(ind))

if(any(complete.cases(state[,4])&state[,4]=="5"))

dec_sc5 <- probabilities[,which(state[,4]=="5")]*0.253

dec_ua2 <- rep(0,length(ind))

if(any(complete.cases(state[,5])&state[,5]=="2"))

dec_ua2 <- probabilities[,which(state[,5]=="2")]*0.233

dec_ua3 <- rep(0,length(ind))

if(any(complete.cases(state[,5])&state[,5]=="3"))

dec_ua3 <- probabilities[,which(state[,5]=="3")]*0.233

dec_ua4 <- rep(0,length(ind))

if(any(complete.cases(state[,5])&state[,5]=="4"))

dec_ua4 <- probabilities[,which(state[,5]=="4")]*0.233

dec_ua5 <- rep(0,length(ind))

if(any(complete.cases(state[,5])&state[,5]=="5"))

dec_ua5 <- probabilities[,which(state[,5]=="5")]*0.233

dis <- (dec_mo2 + dec_sc2 + dec_ua2 + dec_pd2 + dec_ad2)*0.191 +

(dec_mo3 + dec_sc3 + dec_ua3 + dec_pd3 + dec_ad3)*0.458 +

(dec_mo4 + dec_sc4 + dec_ua4 + dec_pd4 + dec_ad4)*0.832 +

(dec_mo5 + dec_sc5 + dec_ua5 + dec_pd5 + dec_ad5)

eq5d_mv <- full_health + (1 - dis)*(1-full_health)

rm(chol_1b,constraints,inds.cat,levels,list_R,mm,pred.fixedL,pred.fixedU,

pred.lower,pred.upper,predictors,probabilities,response.cat,S,select,

sigma,state,theta,theta.lower,theta.upper,Tz,var_cov_1b,XcatL,XcatU,

coefficients,elements,full_health,i,ind,j,k,l,list_cat_1,list_cat_2,

n,ncat,prob,probabilistic,random.draw,S_full,stddevs,subjectID,dec_ad2,

dec_ad3, dec_ad4, dec_ad5, dec_mo2, dec_mo3, dec_mo4, dec_mo5,

dec_pd2, dec_pd3, dec_pd4, dec_pd5, dec_sc2, dec_sc3, dec_sc4, dec_sc5,

dec_ua2, dec_ua3, dec_ua4, dec_ua5, dis)

### Deterministic example estimates should be (probabilistic ones will

### vary):

### 0.8129452 0.5391561 0.4839176 0.7005031 -0.1113542
